# Participant or bystander: effect of teaching styles on viewer engagement in doctor-generated short videos

**DOI:** 10.3389/fpubh.2026.1782353

**Published:** 2026-03-20

**Authors:** Xin Liu, Fengjuan Han, Ying Shen, Fangyuan Liu

**Affiliations:** 1 School of Management, Harbin Institute of Technology, Harbin, China; 2 Department of Gynecology, The First Affiliated Hospital of Heilongjiang University of Chinese Medicine, Harbin, China

**Keywords:** digital healthcare, knowledge dissemination, short videos, teaching styles, viewer engagement

## Abstract

**Purpose:**

In contemporary society, the effective dissemination of medical knowledge and the sustained improvement of health literacy have become paramount priorities for public health. Fortunately, short video platforms have paved new avenues for doctors to teach medical information and communicate health concepts to the public. Despite their substantial significance, short videos still fall short in eliciting viewer engagement, thereby limiting their meaning. In response, this study aims to assist doctors in leveraging teaching styles to refine the design and creation of short videos.

**Methods:**

Based on data from Douyin (Chinese TikTok), this study employs econometric analyses to examine the influences of two prevalent teaching styles—monologue vs. dialogue—on viewer engagement in short videos, including likes, comments, collections, and shares. Furthermore, our study empirically examines the moderating roles of teaching content characteristics, including knowledge professionalism and knowledge generality, in these influences.

**Results:**

The results demonstrate that monologue significantly outperforms dialogue in terms of viewer engagement, spanning from likes to shares. Moreover, the decrease in knowledge professionalism and the increase in knowledge generality can enhance the advantages of monologue over dialogue.

**Conclusions:**

This study contributes to the understanding of viewer engagement in short videos and guides doctors to generate high-impact short videos, advancing the public's health self-management and medical knowledge dissemination.

## 1 Introduction

The improvement in living standards promotes public health consciousness (1, 2). In an era characterized by escalating health demands, severe challenges have been posed to public health by potential threats, including infectious diseases (e.g., COVID-19 pandemic), medical emergencies (e.g., myocardial infarction), and chronic conditions (e.g., diabetes) (3–5). As the contradiction between health demands and health threats intensifies, people's health ideas have changed from a “dependent” to a “self-help” approach, which fosters an awareness of health self-management (6, 7). In this context, short video platforms, represented by Douyin (Chinese TikTok), have emerged as prominent avenues for facilitating medical and health (M&H) knowledge dissemination from healthcare professionals to the public (8, 9). Due to a broad user base and strong influence, short videos contribute to the realization of knowledge dissemination within the healthcare domain (10–13).

Douyin receives 21,000 new doctor-generated short videos daily and provides content relevant to healthcare to 200 million users (https://www.douyin.com, 2023). These short videos offer accessible resources to meet social demands for M&H knowledge (14–16). On short video platforms, viewers can interact with creators through forms of viewer engagement, including liking, commenting, collecting, and sharing, to express their support, share their own feedback, revisit M&H information, or further spread information to others in need. Viewer engagement can be regarded as an indicator of the potential reach of and initial interest in short videos (11, 17, 18). On the one hand, as viewer engagement reflects interactions from individuals who have been exposed to the content, higher engagement may suggest a greater capacity for knowledge diffusion. Specifically, the platform's recommendation algorithm pushes short videos that receive more viewer engagement to more viewers, which enhances M&H knowledge visibility. Besides, through sharing, viewers transmit knowledge to a broader group. On the other hand, viewer engagement, such as likes and comments, indicates the interest of viewers. For instance, through commenting, viewers can express their feeling of viewing short videos, which mobilizes their cognitive resources related to their interests. Meanwhile, they utilize collections to view the videos they are interested in repeatedly. Although viewer engagement does not necessarily imply comprehension, retention, or the ultimate social impact of the M&H knowledge, it is helpful to prompt health communication and education (10, 19). Notably, due to professional constraints, doctors often lack expertise in the creation of short videos, resulting in suboptimal viewer engagement and even no viewer engagement (20). From the perspective of viewer engagement, the efforts of doctors for M&H knowledge dissemination have not yet generated their full value.

**Figure 1:**
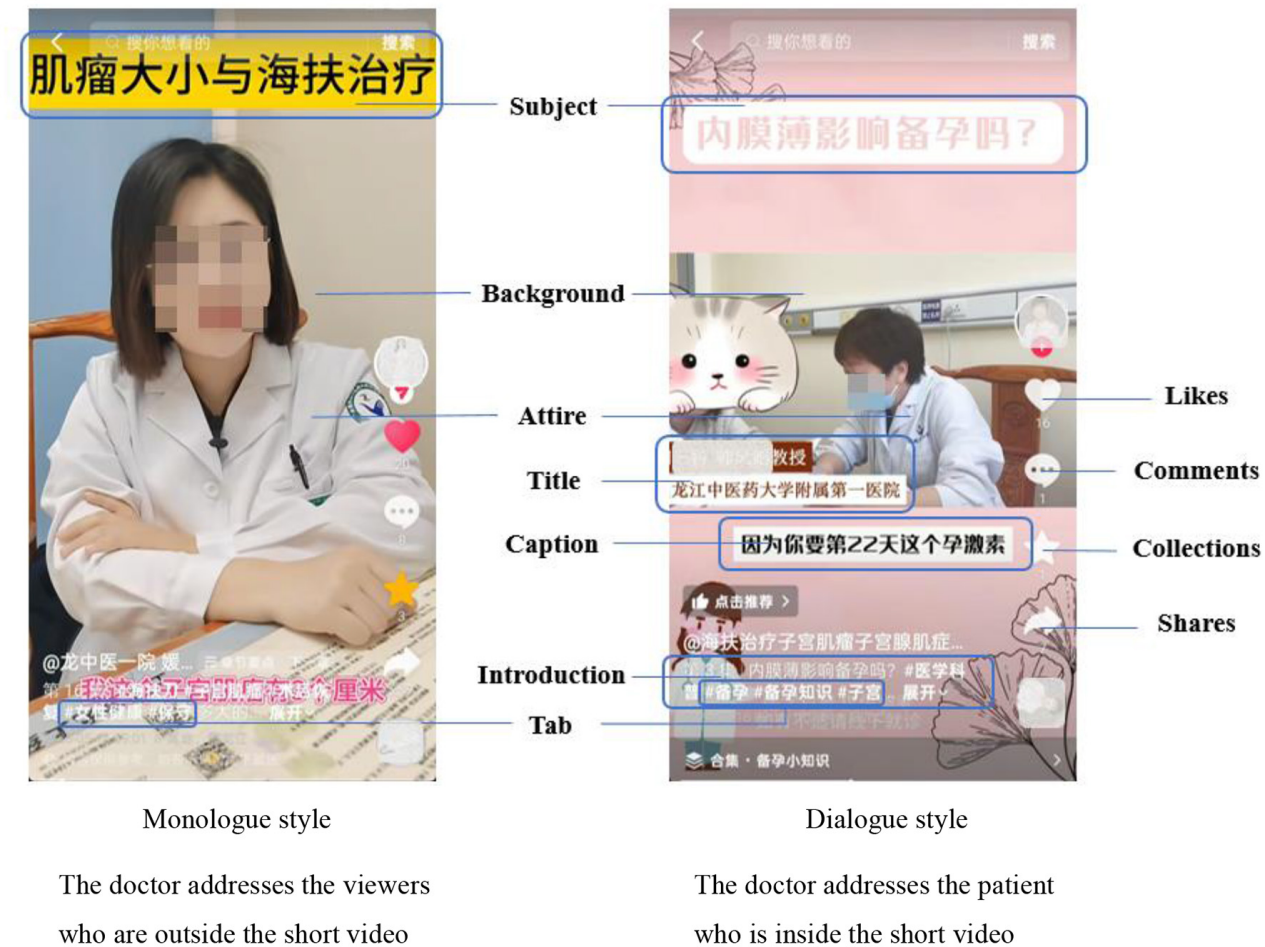
Teaching styles in doctor-generated short videos.

Previous studies have discussed strategies to enhance viewer engagement by optimizing various aspects of doctor-generated short videos (19, 21–24). They tagged short videos with basic features (e.g., video length), visual features (e.g., the doctor's image), and auditory features (e.g., background music). On this basis, these studies have examined the factors related to viewer engagement, while omitting to explore the effect of teaching styles. According to Social Exchange Theory (SET) (25, 26), viewers are willing to interact because they perceive the value of short videos, including M&H knowledge. The perceived value may be contingent upon teaching styles adopted by doctors in their short videos (27–29). As shown in Figure 1, two predominant teaching styles in doctor-generated short videos, monologue and dialogue, may evoke different viewers' feelings about health communication and satisfy their knowledge needs to varying degrees, which influence their perceived value and consequently viewer engagement (28).

The systematic integration of teaching styles with teaching content serves as a determinant of instructional effectiveness and learners' perceived value (30, 31). It is consequently important to know how teaching content moderates the effect of teaching styles on viewer engagement. However, previous research has not sufficiently explored this topic. In the field of M&H knowledge dissemination, two characteristics of teaching content deserve attention: knowledge specialization and knowledge generality (32, 33). Knowledge specialization represents the reason for conducting advanced tiers of medical education (34). Although systematic and in-depth knowledge advances doctors' understanding of the medical domain, the public, when acting as learners, is unable to assimilate it easily because of a lack of theoretical foundation and comprehension capacity. Knowledge generality reflects health conditions or pathological states that exhibit a high incidence rate or are widespread within a population (35). Due to their generality, numerous viewers are eager to learn the relevant knowledge. As such, knowledge specialization and generality may act upon the effect of teaching styles on viewer engagement.

We conduct our research in three aspects based on over 2,400 doctor-generated short videos. First, we analyze the effect of teaching styles on viewer engagement, ranging from likes to shares. Second, this study investigates the moderating role of knowledge specialization in the effect. Third, our research examines whether knowledge generality moderates the effect. The findings reveal that the monologue style brings about greater viewer engagement compared with the dialogue style. A decrease in knowledge specialization and an increase in knowledge generality heighten this effect. Our study enriches theoretical literature on M&H knowledge dissemination and offers practical suggestions for improving medical knowledge dissemination and health communication by doctor-generated short videos.

## 2 Literature review

### 2.1 M&H knowledge dissemination

M&H knowledge is a systematic collection of scientific theories and empirical conclusions associated with human health maintenance, disease prevention, diagnostic checks, treatment strategies, and rehabilitation management (36). It spans the whole range from basic research to clinical applications and is rooted not only in the long-term accumulation of medical practice but also in the interdisciplinary integration with advancements in biology, chemistry, physics, and related fields. Notably, the highly specialized nature of medicine creates significant barriers to disseminating knowledge to target audiences such as the patient population and populations susceptible to disease, resulting in the insufficient utilization of M&H knowledge (37). This situation highlights the need for healthcare professionals to engage in public education. Consequently, M&H knowledge dissemination has emerged as a strategic measure to enhance national health literacy and advance disease prevention systems (38). Due to the advantages of short videos, such as visual presentation, fragmented dissemination, and strong interactivity, the knowledge socialization implemented by healthcare professionals through short video platforms has become the preferred approach for M&H knowledge dissemination (12).

M&H short videos cover aspects of nutritional health, disease prevention, first aid knowledge, and clinical treatment. Although these short videos provide rich knowledge to viewers, their information quality varies significantly (39–42). Through DISCERN-based evaluations by professionals, Song et al. (43) found that the knowledge quality of short videos relevant to chronic obstructive pulmonary disease depends on the videos' sources, where non-profit organizations, news agencies, and healthcare professionals offer higher-quality knowledge than for-profit organizations and general users. He et al. (44) demonstrated that short videos related to inflammatory bowel disease created by professionals are of higher quality than those produced by non-professionals. Gong et al. and Zhang et al. reported similar results regarding coronary heart disease and cervical cancer (45, 46). These findings confirm that the content created by healthcare professionals ensures the knowledge quality of M&H short videos. Additionally, short video platforms also play a similar role; for instance, M&H short videos are of superior knowledge quality on TikTok compared to Kwai (46). When watching these high-quality short videos, viewers can obtain useful information, which enhances the effectiveness of knowledge dissemination (1). Previous studies have generally employed some mature scales, such as DISCERN and JAMA criteria, to conduct assessments, whereas they have paid less attention to knowledge specialization and generality (4, 43–45).

Since viewers are highly sensitive to video content and elements, including creator characteristics and special effects, their strategic application has become a research topic (13, 19, 24, 47). While video content and elements are not directly associated with educational objectives, their entertainment value could potentially increase viewer engagement and attention span. However, misuse may pose a risk of cognitive distraction and information fragmentation (13, 47). In this regard, Tan et al. (19) identified doctors' on-camera attire, refined looks, and title displays as factors that enhance viewer engagement. Zhang W et al. (47) suggested that viewers prefer M&H short videos with movie clips and obvious subtitles. Background music, progress bars, durations under 60 s, and official language are verified as useful elements to promote viewer engagement in M&H short videos (13, 24). By contrast, teaching styles exhibited in short videos remain underexplored in previous studies, which motivates our study to focus on teaching styles.

### 2.2 Viewer engagement

In the online environment, engagement describes interactive activities in the processes of information dissemination (48). User engagement represents a multidimensional concept from cognitive to emotional aspects, where behaviors serve as the most direct manifestation (49). On short video platforms, user engagement can be categorized based on user types. With respect to creators, engagement includes short video generation, uploading, and deletion (50). With respect to viewers, engagement primarily emerges through watching, liking, commenting, collecting, and sharing (19). In the field of M&H short videos, scholarly attention mainly revolves around viewer engagement, as it serves as the objective of creator engagement while reflecting the results of knowledge dissemination. Previous research has indicated that factors tied to viewer engagement span a broad range, from the characteristics of creators to those of viewers (19, 51, 52). Alongside these factors, researchers have proposed that viewer engagement is rooted in its underlying psychological mechanisms, such as social exchange motivation and reciprocity motivation (52, 53).

Social exchange motivation, as a core concept of social exchange theory, is used to elucidate the intrinsic mechanism driving social behaviors (25). Grounded in the economic input-output model, social exchange theory posits that human social interactions fundamentally lie in exchange processes of informational, emotional, and economic resources, and that social relationships are inherently social exchange relationships (26). While economic exchanges require tangible benefits, social exchanges aim to obtain intangible returns, such as psychological satisfaction. Within the framework of this theory, commitment, loyalty, and trust are regarded as outcomes of long-term social relationships, which require adherence to norms such as reciprocity and altruism in interpersonal interactions (25). Under these norms, the recipients of benefits feel obligated to reciprocate the providers of benefits (54). Due to the obtained benefits and anticipated returns, individuals are motivated to sustain relationships through mutually beneficial behaviors. Xu et al. (53) utilized social exchange motivation to illustrate that individuals are more willing to participate in knowledge sharing when they obtain richer M&H knowledge on short video platforms.

## 3 Hypothesis development

### 3.1 The effect of teaching styles

According to SET, after acquiring benefits, individuals tend to reciprocate the actions of another party in social interactions (25, 26). Accordingly, viewers are more inclined to engage in reciprocal behaviors to reinforce bonds with creators and other viewers when they perceive more value in M&H short videos. The accumulation of likes, comments, collections, and shares reflects viewer approval and support, which creates a positive feedback loop between creators and viewers (11, 14, 19, 53). First, viewers prefer to like short videos that are aligned with their needs. Through giving likes, viewers respond to the efforts of creators, which fosters an online relationship grounded in mutual contribution. Second, viewers actively comment on such short videos. Posting comments enables viewers to express emotions, share opinions, and exchange experiences with both creators and other viewers, facilitating interactive communication. Third, as a method of memory management, collections allow viewers to archive these short videos for future re-visitation. Beyond facilitating repeated access, collecting sustains long-term connections with creators by representing enduring interest and affiliation. Last, since shares can help others acquire knowledge or address needs, sharing extends the potential reach of M&H short videos. Shares not only amplify viewer engagement to enable social exchange between creators and viewers but also enhance social bonds among viewers by increasing collective welfare. Viewer engagement manifested by these behaviors reinforces relational capital and value loops between viewers and creators (11, 20).

Viewer engagement depends on the perceived value of M&H short videos by viewers, where the perceptual process involves processing information of both simple and complex cues (55). The Heuristic–Systematic Model (HSM), a well-known model of information processing, posits that people use dual processes when processing information, including heuristic and systematic processing (56). Heuristic processing functions as an efficient, automatic mode for rapidly assimilating simple cues, whereas systematic processing is reserved for complex cues that demand focused attention, deliberate contemplation, and effortful reasoning (57).

When watching M&H short videos, simple cues that viewers can directly observe and intuitively judge include teaching styles, such as monologue and dialogue, which invoke the heuristic process. In the monologue style, where doctors directly address viewers through the screen, viewers experience a high sense of participation, as these short videos are presented as personalized guidance tailored for them by doctors (58). By contrast, the dialogue style shows clinical consultations between doctors and patients, which situates viewers as passive observers. Additionally, doctors who adopt the monologue style strategically align their explanations with the knowledge needs of the general population based on clinical epidemiology data, with script refinement and appearance preparation. Conversely, due to the demonstration of personalized case analysis and treatment recommendations tailored to specific patients, dialogue-style short videos prioritize individual case discussions over systematic knowledge dissemination (59). These clinical consultations also place less emphasis on the preparation of scripts and appearances. Given these differences, viewers may consider monologue-style short videos more helpful, specifically for meeting their needs for M&H knowledge, thereby fostering their engagement for reciprocity in the social exchange relationship. Therefore, we posit that the monologue style can promote viewer engagement, as follows:

Hypothesis 1: Compared with the dialogue style, the monologue style elicits greater viewer engagement in M&H short videos.

### 3.2 The moderating role of knowledge specialization

HSM indicates that when facing information, individuals utilize systematic processing to understand complex cues while integrating these outcomes with those from heuristic processing relevant to simple cues (57, 60). M&H knowledge systems are inherently intricate and hierarchical, which presents learning barriers for non-expert populations (34). Therefore, the specialization of teaching content in M&H short videos can be considered a complex cue and requires viewers to use systematic processing to understand.

When doctors explain highly specialized content, viewers with limited comprehension capacities in the M&H domain absorb less knowledge because of cognitive overload. Conversely, simplified introductions that bridge professional knowledge with public understanding enhance viewers' knowledge assimilation. Based on HSM, knowledge specialization (a complex cue) can interact with teaching styles (a simple cue) to influence viewers' perceived value of M&H short videos, which in turn determines viewer engagement (61). Specifically, monologue-style short videos provide viewers with more perceived value compared with dialogue-style short videos. As knowledge specialization increases, viewers' perceived value of monologue-style short videos can significantly decrease due to a higher cognitive threshold and reduced knowledge acquisition. In contrast, dialogue-style short videos inherently demonstrate low perceived value, which renders the negative effect of knowledge specialization comparatively less pronounced. Consequently, increased knowledge specialization reduces the comparative advantage of the monologue style over the dialogue style in terms of viewer engagement. This leads to the following hypothesis:

Hypothesis 2: As knowledge specialization increases, the positive effect of the monologue style (relative to the dialogue style) on viewer engagement decreases.

### 3.3 The moderating role of knowledge generality

Knowledge generality is another complex cue in M&H short videos (35). Viewers watch M&H short videos with the primary goal of improving their knowledge reserves, thereby enhancing their future ability to address health-related issues or optimize outcomes after treatment (8, 43). For different topics in medicine and healthcare, the applicability of M&H knowledge varies significantly (62). Frequently encountered ailments, such as the common cold, occur multiple times, whereas relatively rare conditions, such as AIDS, are beyond the life experiences of most people. When doctors present knowledge with high generality in their short videos, viewers are more likely to apply this knowledge in subsequent practical scenarios. Conversely, content addressing rare diseases holds minimal relevance for the majority of viewers. As knowledge generality increases, monologue-style short videos offer greater practical application and public health benefits, which substantially amplify viewers' perceived value. However, dialogue-style short videos offer insufficient perceived value, a benefit that is difficult to enhance even with an increase in knowledge generality. Consequently, increased knowledge generality amplifies the advantage of the monologue style compared to the dialogue style in terms of viewer engagement. The hypothesis is formally formulated:

Hypothesis 3: As knowledge generality increases, the positive effect of the monologue style (relative to the dialogue style) on viewer engagement increases.

### 3.4 Theoretical framework

Viewer engagement stems from a continuous process from information content to perceived value and then to behavioral feedback, wherein HSM and SET play complementary and mutually embedded roles throughout this process.

Firstly, HSM elucidates how viewers form the perceived value from information content. According to HSM, viewing short videos engages two cognitive processing modes, heuristic and systematic, that collaboratively process the information of short videos. On the one hand, teaching styles (monologue and dialogue) serve as typically simple cues, rapidly triggering heuristic processing. Viewers may judge the potential significance of the short video based solely on visual experience, thereby forming an initial perceived value. On the other hand, as the video content unfolds, the teaching content itself serves as a core complex cue, which activates systematic processing. Viewers deeply evaluate the specialization and generality of the content, thus refining their initial value judgments. The dynamic interaction of these two processing modes shapes viewers' perceived value. Without the information processing mechanism described by HSM, viewers would be unable to derive benefit from the audiovisual experience of short videos, rendering the act of viewing itself meaningless.

Secondly, SET reveals how perceived value translates into behavioral feedback. SET conceptualizes the interaction between viewers and creators as a social exchange relationship. When viewers perceive sufficient value (e.g., useful knowledge) from short videos through the information processing outlined by HSM, they will develop a motivation to reciprocate. This motivation manifests as viewer engagement, such as likes and comments, constructing a positive feedback loop between viewers and creators, thereby sustaining the beneficial social relationship. Without this motivation, as explained by SET, viewers would lack the impetus to take practical action, even when engagement costs are minimal (e.g., a simple click for likes).

The complementarity of the two theories lies in the former explaining “how value is perceived from the video” and the latter explaining “how perceived value drives subsequent behaviors.” Together, they address the core question of “how information facilitates behaviors.” A single theory cannot fully explain the entire process from information reception to behavior generation: HSM stops at cognition, failing to extend to behaviors; SET presupposes the existence of value but does not reveal how value is dynamically constructed during information processing. Their combination precisely forms a complete explanatory chain.

Furthermore, this study has not focused on other theoretical frameworks, such as Cognitive Load Theory, Parasocial Interaction Theory, and Narrative Persuasion Theory, for the following reasons. First, Cognitive Load Theory suggests that information presentation and content structure affect learners' cognitive resource allocation (intrinsic, extraneous, and germane load), thereby influencing learning outcomes. However, in this study, while teaching content might involve cognitive load, teaching styles are weakly related to the core concepts of cognitive load, and this theory is also inadequate for revealing the interactive effects produced by different information. Second, Parasocial Interaction Theory posits that media figures can create scenarios resembling real interaction with viewers through certain presentational styles, thereby influencing viewers' feedback. Although monologue and dialogue could be interpreted as such presentational styles, this theory fails to encompass viewers' rational evaluation of teaching content itself. Finally, Narrative Persuasion Theory applies to content with strong narrative elements, emphasizing the impact of plot and narrative structure. However, doctor-generated short videos contain substantial non-narrative elements like knowledge explanations and skill demonstrations, limiting this theory's applicability. Additionally, as visual material, monologue and dialogue align more closely with the concept of HSM rather than the domain of Narrative Persuasion Theory.

**Figure 2:**
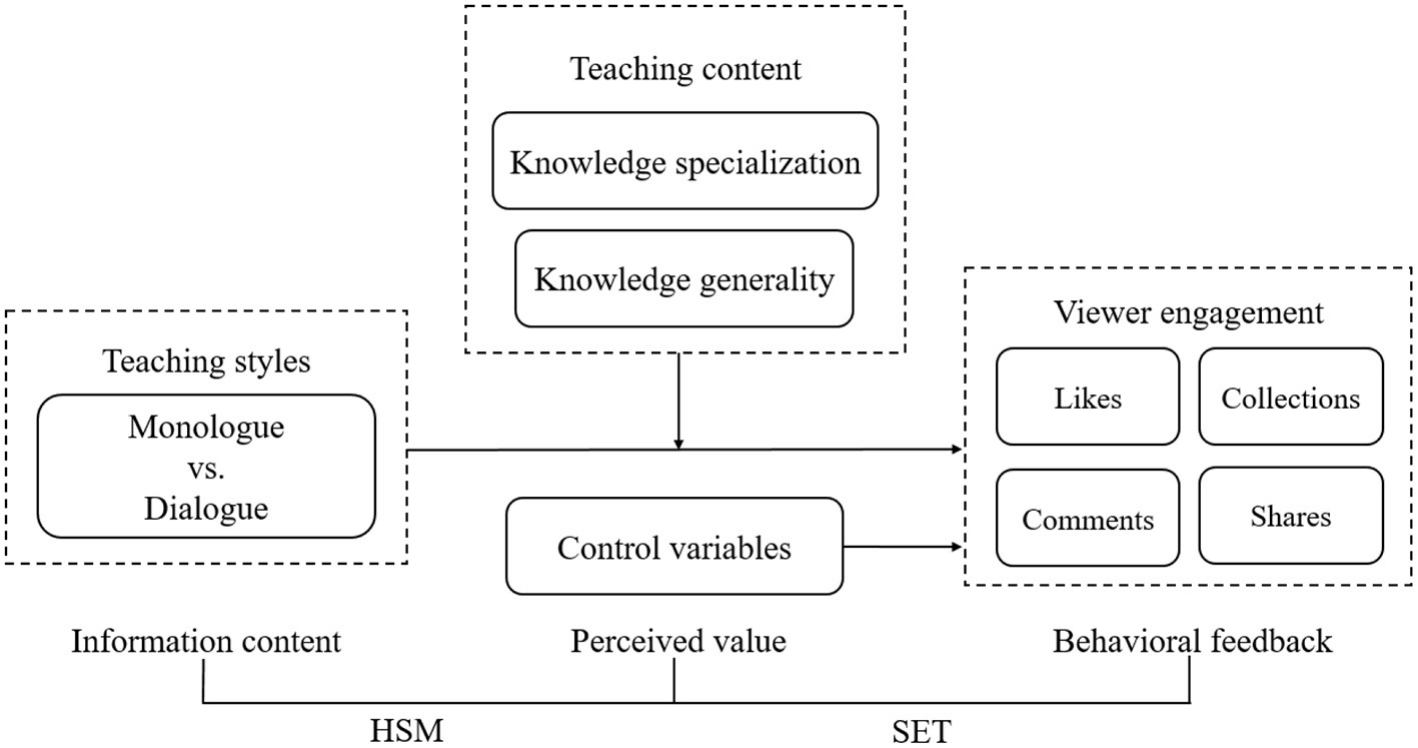
Theoretical framework of the present study.

Figure 2 shows the theoretical framework of the present study.

## 4 Methods

### 4.1 Data

This study collected data from M&H short videos on Douyin, one of the largest user-generated short video platforms globally, with over one billion users (9). We searched Douyin videos using several keywords, including “医生” (doctor), “医生问诊” (doctor consultation), “医疗科普” (medical science popularization), and “健康科普” (health science popularization). Subsequently, we identified the top 100 Douyin-verified doctors as candidates. Among these candidate doctors, we selected those who had released both types of M&H short videos—monologue and dialogue—from January to May 2024. These short videos constituted our research sample. Finally, we obtained 2,412 short videos sourced from 30 doctors. Notably, all short videos were produced by certified doctors, all of whom are officially certified by national authorities (doctors provide qualification certificates). Moreover, every uploaded video was subject to the platform's internal review process to ensure compliance with community guidelines. The knowledge presented in these short videos covers a wide range of medical disciplines, such as internal medicine, surgery, gynecology, and pediatrics. Short videos posted by the same doctor may impart knowledge across different medical disciplines, even if the topics fall outside the doctor's primary specialization.

### 4.2 Variable measurement

Drawing upon previous research, we utilized two approaches to identify video features (19, 43). First, the objective features of short videos are easily observable and classifiable by viewers, such as teaching styles and backgrounds. Given their identifiability, one doctoral candidate was assigned to select these features based on the coding instructions shown in Table 1 (labeled as “objective” in the “Category” column) (19). One clinician determined the medical topic to which the knowledge presented in the short video belonged.

**Table 1:** The coding instructions for video features.

**Variable**	**Category**	**Data type**	**Score rule**
**Independence variable**			
Style	Objective	Categorical	Which teaching style is used: Monologue = 0, Dialogue = 1
**Moderating variables**			
Special	Subjective	Ordinal (1–6 points)	From 1 to 6, knowledge specialization gradually increases. Specifically: 1 = Completely life-oriented, requiring no medical background. The content can be fully understood through everyday life experiences or common sense accessible to any layperson. 2 = Primarily life-oriented, with basic health concepts. The content uses common health terms that have entered everyday discourse and can be understood through life experience and common sense. 3 = Slightly life-oriented, with popularized professional terms. The content incorporates some professional terms that have become common in everyday health discourse. These terms are recognizable to the public and can be understood with basic medical common sense. 4 = Slightly profession-oriented, with explained terminology. The content introduces professional terms that the public infrequently encounters. Although medical terminology is used, the content provides simple explanations or metaphors. After minimal reflection, viewers with basic medical common sense can grasp the meaning. 5 = Mainly profession-oriented, with unexplained terminology. The content relies on professional terms that are not easy to simplify or illustrate. Medical terminology is used without adequate explanation or lay-friendly simplification, requiring specialized medical expertise to fully understand. 6 = Completely profession-oriented, requiring expert knowledge. The content presents highly specialized knowledge, such as recent clinical theories, pathophysiological mechanisms, or philosophical dimensions of medicine. The terminology is not amenable to simplification, and even individuals with general medical expertise may find it challenging.
General	Subjective	Ordinal (1–6 points)	From 1 to 6, knowledge generality gradually increases. Specifically: 1 = Highly specialized, minimal public exposure. The topic involves diseases or conditions that are very rare in the general population or can be a shock. The public may have heard the name, but has almost zero chance of direct personal or vicarious (through acquaintance) experience. Examples: leukemia, AIDS. 2 = Specialized, occasionally clinical exposure. The topic covers health issues that are specialized and primarily encountered in specific healthcare settings. The public may recognize the term, but personal encounters are very infrequent. Examples: specific cancers, complex contagions. 3 = Clinically common, limited public contact. The topic involves conditions that are common in clinical practice but often remain abstract or unseen in daily life. The public knows they exist, but they are usually not a topic of daily conversation or personal worry for the average healthy person. Examples: organ failure, cerebral hemorrhage. 4 = Daily relevant, moderate public contact. The topic deals with health issues that a significant portion of the public will personally encounter or know someone who has. These are common reasons for visiting a doctor and are often discussed in public. Examples: hypertension, diabetes. 5 = Highly prevalent, strong public health relevance. The topic covers conditions that are ubiquitous and regularly experienced by nearly everyone. They are a common part of human life, requiring frequent self-management or basic first aid. Examples: common cold, indigestion. 6 = Fundamental health topics, universal applicability. The topic is about fundamental health conditions that apply to everyone, every day. It is not only about treating an illness, but about maintaining health, and is embedded in a routine lifestyle. These concepts require no medical context. Examples: nutrition fundamentals, hygiene practices.
**Dependent variables**			
Likes		Numeric	Same as video statistics.
Comments		Numeric	Same as video statistics.
Collections		Numeric	Same as video statistics.
Shares		Numeric	Same as video statistics.
**Control variables**			
Background	Objective	Categorical	Which scenario serves as the background:
			Home = 0, Hospital office = 1, Hospital corridor = 2, Outdoors = 3, Wall = 4
Attire	Objective	Categorical	Which clothes does the doctor wear: Casual clothes = 0, White uniform = 1, Scrubs= 2
Appearance	Objective	Categorical	How many people appear: One people = 0, Two people = 1, More people = 2
Range	Objective	Categorical	How is the doctor presented: Headshot = 0, Bust shot = 1, Full body shot = 2
Angle	Objective	Categorical	How does the doctor face the camera: Straight = 0, Lateral = 1
			Does it have the following elements (None = 0, Have = 1)
Introduction	Objective	Categorical	A brief for the video content in the bottom left area
Subject	Objective	Categorical	A brief for the subject in the top area
Cover	Objective	Categorical	A description of the video content in the doctor's video list
Sticker	Objective	Categorical	Visual elements for artistic expression, such as emoticons and animations
Insert	Objective	Categorical	Visual elements for explaining content, such as picture-in-picture and embedded videos
Title	Objective	Categorical	A description of the doctor's title
Music	Objective	Categorical	Auditory elements for video atmosphere
Tab	Objective	Categorical	Links directed to the search results of the text in the Introduction
Caption	Objective	Categorical	The subtitle of the monologue or the dialogue
Distance	Objective	Numeric	Temporal distance between video release and our observation (days)
Length	Objective	Numeric	Duration of short video (seconds)
Topic	Subjective	Categorical	The categorization of the medical knowledge among cardiology, gynecology, etc.

Second, two clinicians from a tertiary Grade A teaching hospital, who were invited to participate in our study, used a 6-point scale to score subjective features of short videos based on medical guidelines and clinical experience, including knowledge specialization and generality. Compared with other common scales, such as the 5-point scale, the 6-point scale performs well in terms of skewness and normality, while avoiding the effects of a neutral point (63). During a face-to-face meeting, we explained to the clinicians how to encode these features, and they confirmed their full understanding of the rating instructions presented in Table 1 (labeled as “subjective” in the “Category” column). With regard to construct validity, we invited five doctors with rich clinical experience to assess the relevance of each rating instruction to its corresponding construct. They rated the instructions using a 4-point scale (1 = not relevant, 2 = somewhat relevant, 3 = quite relevant, 4 = highly relevant). The I-CVI scores for knowledge specialization and generality were 1.00, and the S-CVI was also 1.00, confirming the adequacy of the rating instructions. With respect to the reliability of subjective assessments, the consistency of the scores provided by two clinicians was assessed using the linearly weighted kappa coefficient (19, 64). The results for knowledge specialization (κ = 0.735, 95% CI: 0.717–0.754) and knowledge generality (κ = 0.811, 95% CI: 0.797–0.826) indicated high consistency in their evaluations.

Table 2 lists the descriptive statistics of variables: Part 1 provides details of numeric and ordinal variables, while Part 2 summarizes categorical variables. It can be observed that 59.7% (*n* = 1,441) of the videos are presented in a monologue style, while 40.3% (*n* = 971) are structured as dialogues. Additionally, the average length of these short videos is 57 s (standard deviation is 36), with a minimum of 6 s and a maximum of 370 s.

**Table 2:** Descriptive statistics of variables.

**Part 1: Numeric and ordinal variables**					
**Variable**	**Number**	**Min**.	**Max**.	**Average**	**Standard deviation**
**Control variables**					
Distance	2,412	0	200	109.88	36.993
Length	2,412	6	370	56.717	36.155
**Moderating variables**					
Special	2,412	1	6	3.2763	0.9753
General	2,412	1	6	4.3864	1.4306
**Dependent variables**					
Likes	2,412	13	1,322,027	10,914.3	44,379.7
Comments	2,412	0	167,571	676.15	4,763.4
Collections	2,412	0	622,890	3,648.0	18,986.6
Shares	2,412	0	1,217,389	5,285.4	36,746.1
**Part 2: Categorical variables**						
**Variable**	**Number (Percentage)**				
**Independence variable**						
Style	Monologue = 1,441 (59.7%), Dialogue = 971 (40.3%)				
**Control variables**						
Background	Home = 329 (13.6%), Hospital office = 1,668 (69.2%), Hospital corridor = 54 (2.2%), Outdoors = 32 (1.3%), Wall = 329(13.6%)				
Attire	Casual clothes = 157 (6.5%), White uniform = 2,121 (87.9%), Scrubs = 134 (5.5%)				
Appearance	One people = 2,150 (89.1%), Two people = 180 (7.5%), More people = 82 (3.4%)				
Range	Headshot = 1,891 (78.4%), Bust shot = 412 (17.1%), Full body shot = 109 (4.5%)				
Angle	Straight = 1,495 (62.0%), Lateral = 917 (38.0%)				
Introduction	None = 505 (20.9%), Have = 1,907 (79.1%)				
Subject	None = 1,384 (57.4%), Have = 1,028 (42.6%)				
Cover	None = 177 (7.3%), Have = 2,235 (92.7%)				
Sticker	None = 1,133 (47.0%), Have = 1,279 (53.0%)				
Insert	None = 1,966 (81.5%), Have = 446 (18.5%)				
Title	None = 836 (34.7%), Have = 1,576 (65.3%)				
Music	None = 491 (20.4%), Have = 1,921 (79.6%)				
Tab	None = 60 (2.5%), Have = 2,352 (97.5%)				
Caption	None = 462 (19.2%), Have = 950 (80.8%)				

### 4.3 Model specification

For the effect of teaching styles, we established the following equation:


$$\begin{array}{l} {\text{Υ}}_{i}=\beta_{0}+\beta_{1}{Style}_{i}+\varphi X_{i}+\mu +\omega +\varepsilon_{i} \end{array}$$

where Υ represents the number of likes, comments, collections, and shares of short video ***i***; ***X*** is the set of control variables; **μ** expresses the individual-fixed effects (doctor-level), and **ω** signs the time-fixed effects (month-level) to account for the doctor-related and temporal heterogeneity; **ε** serves as the random error term.

For the moderating effects, we added the interaction items between the independent variable and the moderating variables in the above equation:


$$\begin{array}{l} {\text{Υ}}_{i}=\beta_{0}+\beta_{1}{Style}_{i}+\beta_{2}{Special}_{i}\\ \;+\delta_{1}{Style}_{i}\times {Special}_{i}+\varphi X_{i}+\mu +\omega +\varepsilon_{i}\\ {\text{Υ}}_{i}=\beta_{0}+\beta_{1}{Style}_{i}+\beta_{2}{General}_{i}+\delta_{1}{Style}_{i}\times {General}_{i}+\varphi X_{i}\\ \;+\mu +\omega +\varepsilon_{i}\\ {\text{Υ}}_{i}=\beta_{0}+\beta_{1}{Style}_{i}+\beta_{2}{Special}_{i}+\beta_{3}{General}_{i}+\delta_{1}{Style}_{i}\\ \;\times {Special}_{i}\\ \;+\delta_{2}{Style}_{i}\times {General}_{i}+\varphi X_{i}+\mu +\omega +\varepsilon_{i} \end{array}$$

Building on previous studies, we conducted regression analyses using two distinct modeling approaches. First, as these dependent variables are essentially count variables, we estimated a Poisson regression model with multidimensional fixed effects (Model 1 for count variables) (65, 66). Second, in some prior studies, these variables have been treated as continuous and have therefore been log-transformed (67, 68). Accordingly, we employed an ordinary least squares regression model with multidimensional fixed effects (Model 2 for continuous variables). To mitigate skewness and heteroscedasticity, we log-transformed several control variables, including temporal distance and video length. The variance inflation factors of all explanatory variables are less than 4, and the tolerance values are more than 0.3, indicating that multicollinearity is not a concern.

It is noted that although our data were collected at a single point in time, the nature of short video platforms like Douyin introduces a temporal dimension that strengthens causal inference from regression analysis. Importantly, viewer engagement (i.e., likes, comments, collections, and shares) can only occur after the video content has been presented. This temporal precedence, where the independent variable (teaching style) precedes the dependent variables (e.g., likes), provides a necessary condition for causality and mitigates concerns about reverse causality.

## 5 Results

### 5.1 Main results

**Figure 3:**
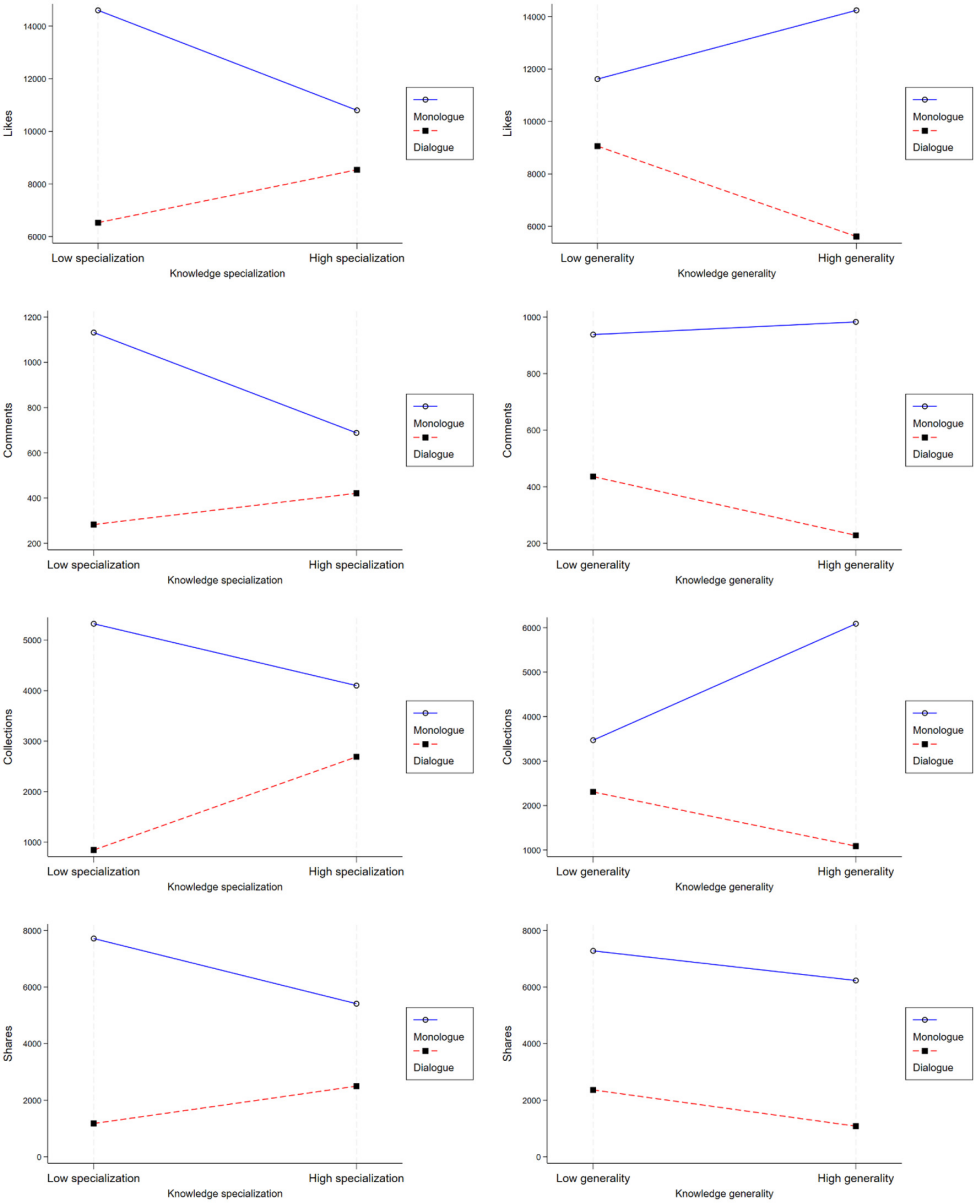
The moderating effects of knowledge specialization and generality.

Table 3 reports the results of the regression analysis. R^2^ values indicate that our explanatory variables collectively explain over 65% of the variance in each dependent variable. In Column (1), the significantly negative coefficients of the independent variable illustrate that doctor-generated short videos elicit less viewer engagement when showing the dialogue style than when showing the monologue style (for likes, β = −0.6282, *p* < 0.05; comments, β = −1.1793, *p* < 0.05; collections, β = −1.3578, *p* < 0.05; shares, β = −1.7561, *p* < 0.05). Thus, Hypothesis 1 is supported. As shown in Column (2), the significantly positive coefficients of the interaction term reveal that the advantage of the monologue style decreases as professional knowledge increases (for likes, β = 0.1435, *p* < 0.05; comments, β = 0.3762, *p* < 0.05; collections, β = 0.4632, *p* < 0.05; shares, β = 0.5546, *p* < 0.05). These results provide support for Hypothesis 2. Column (3) verifies that an increase in knowledge generality can enhance this positive relationship between the monologue style and viewer engagement (for likes, β = −0.2035, *p* < 0.05; comments, β = −0.1630, *p* < 0.05; collections, β = −0.3643, *p* < 0.05; shares, β = −0.0869, *p* > 0.05). Overall, Hypothesis 3 can be confirmed. The Column (4) offers consistent results. Additionally, Model 2 provides more evidence on such findings. Based on Model 1, Figure 3 shows these moderating effects.

**Table 3:** Regression results of viewer engagement.

**Variable**	**Model 1**				**Model 2**			
	**(1)**	**(2)**	**(3)**	**(4)**	**(5)**	**(6)**	**(7)**	**(8)**
**Likes**								
Style	−0.6282^*^ (0.308)	−0.5951^*^ (0.260)	−0.6464^*^ (0.307)	−0.5597^*^ (0.231)	−0.3983^*^ (0.115)	−0.3732^*^ (0.111)	−0.3930^*^ (0.118)	−0.3622^*^ (0.115)
Special		−0.1076 (0.091)		−0.1836^*^ (0.065)		−0.0809 (0.050)		−0.0908 (0.069)
Style × Special		0.1435^*^ (0.070)		0.3469^*^ (0.114)		0.1943^*^ (0.077)		0.2415^*^ (0.108)
General			0.0259 (0.063)	0.0969 (0.055)			−0.0222 (0.040)	0.0115 (0.050)
Style × General			−0.2035^*^ (0.081)	−0.3250^*^ (0.101)			−0.0663 (0.044)	−0.1211^*^ (0.056)
Other variables	Yes	Yes	Yes	Yes	Yes	Yes	Yes	Yes
Cons.	9.2712^*^ (1.497)	9.0041^*^ (1.696)	9.3994^*^ (1.492)	9.2099^*^ (1.582)	6.9417^*^ (0.695)	6.9344^*^ (0.669)	6.9392^*^ (0.704)	6.9576^*^ (0.671)
Observations	2,410	2,410	2,410	2,410	2,410	2,410	2,410	2,410
(Pseudo) R^2^	0.7765	0.7779	0.7780	0.7813	0.8141	0.8150	0.8147	0.8158
**Comments**								
Style	−1.1793^*^ (0.535)	−1.0647^*^ (0.446)	−1.1667^*^ (0.534)	−1.0053^*^ (0.414)	−0.3004^*^ (0.102)	−0.3017^*^ (0.104)	−0.2804^*^ (0.104)	−0.2703^*^ (0.105)
Special		−0.2863^*^ (0.084)		−0.3028^*^ (0.106)		−0.1855^*^ (0.066)		−0.1613^*^ (0.064)
Style × Special		0.3762^*^ (0.153)		0.5447^*^ (0.152)		0.2934^*^ (0.093)		0.3132^*^ (0.106)
General			−0.0813 (0.058)	0.0222 (0.074)			−0.0950^*^ (0.039)	−0.0366 (0.034)
Style × General			−0.1630^*^ (0.079)	−0.3310^*^ (0.096)			−0.0219 (0.050)	−0.1018^*^ (0.048)
Other variables	Yes	Yes	Yes	Yes	Yes	Yes	Yes	Yes
Cons.	6.9398^*^ (2.952)	6.3167^*^ (2.590)	6.8554^*^ (2.679)	6.4208^*^ (2.512)	4.7352^*^ (0.547)	4.6952^*^ (0.508)	4.6918^*^ (0.538)	4.7041^*^ (0.501)
Observations	2,410	2,410	2,410	2,410	2,410	2,410	2,410	2,410
(Pseudo) R^2^	0.6831	0.6898	0.6858	0.6920	0.7393	0.7423	0.7416	0.7438
**Collections**								
Style	−1.3578^*^ (0.340)	−1.2255^*^ (0.286)	−1.4073^*^ (0.357)	−1.2002^*^ (0.286)	−0.6784^*^ (0.165)	−0.5468^*^ (0.141)	−0.6959^*^ (0.167)	−0.5559^*^ (0.146)
Special		0.0458 (0.110)		−0.1591 (0.098)		0.1280 (0.072)		0.0766 (0.101)
Style × Special		0.4632^*^ (0.196)		0.8655^*^ (0.226)		0.1357 (0.092)		0.2264 (0.131)
General			0.2062^*^ (0.083)	0.2676^*^ (0.089)			0.0924 (0.050)	0.0694 (0.071)
Style × General			−0.3643^*^ (0.176)	−0.6255^*^ (0.175)			−0.1564^*^ (0.059)	−0.1749^*^ (0.078)
Other variables	Yes	Yes	Yes	Yes	Yes	Yes	Yes	Yes
Cons.	6.0690^*^ (2.054)	6.2102^*^ (2.260)	6.6631^*^ (1.981)	6.4237^*^ (1.910)	5.4907^*^ (0.882)	5.5696^*^ (0.851)	5.5549^*^ (0.893)	5.6153^*^ (0.851)
Observations	2,410	2,410	2,410	2,410	2,410	2,410	2,410	2,410
(Pseudo) R^2^	0.6490	0.6517	0.6586	0.665	0.7459	0.7484	0.7471	0.7494
**Shares**								
Style	−1.7561^*^ (0.345)	−1.5224^*^ (0.270)	−1.6794^*^ (0.337)	−1.4150^*^ (0.252)	−0.7050^*^ (0.175)	−0.6187^*^ (0.170)	−0.6957^*^ (0.178)	−0.5956^*^ (0.174)
Special		−0.2746^*^ (0.070)		−0.2159 (0.119)		−0.0842 (0.077)		−0.0794 (0.089)
Style × Special		0.5546^*^ (0.138)		0.6747^*^ (0.164)		0.3577^*^ (0.119)		0.4008^*^ (0.145)
General			−0.1479^*^ (0.038)	−0.0740 (0.063)			−0.0414 (0.050)	−0.0096 (0.058)
Style × General			−0.0869 (0.098)	−0.2981^*^ (0.133)			−0.0590 (0.060)	−0.1355^*^ (0.065)
Other variables	Yes	Yes	Yes	Yes	Yes	Yes	Yes	Yes
Cons.	6.7161 (3.873)	5.9460 (3.422)	6.3340 (3.487)	5.8454 (3.218)	4.6707^*^ (0.974)	4.6867^*^ (0.935)	4.6579^*^ (0.970)	4.7075^*^ (0.925)
Observations	2,410	2,410	2,410	2,410	2,410	2,410	2,410	2,410
(Pseudo) R^2^	0.6820	0.6882	0.6864	0.6902	0.7341	0.7359	0.7347	0.7368

^*^p < 0.05; Robust standard errors clustered by doctor are in parentheses (); Other variables include control variables and fixed-effect variables.

### 5.2 Robustness checks

We conducted two robustness checks to solidify our findings, the results of which are listed in Columns (1) to (4) of Table 4. First, we applied the Poisson regression model in the main analysis. Since the assumption of Poisson regression is equidispersion, we used negative binomial regression in this part, which is more suitable for overdispersion (69). Columns (1) and (2) of Table 4 provide these results. Second, given that these dependent variables capture distinct dimensions of viewer engagement, these regression equations can be viewed as interrelated projects within the same system, potentially leading to correlated error terms across equations. To address this issue, we employed a system estimation through seemingly unrelated regression with fixed effects, which is more efficient than separate regression estimations (67). The results are shown in Columns (3) and (4) of Table 4. All robustness checks demonstrate consistency with our primary analysis.

**Table 4:** Regression results of robustness and endogeneity checks.

**Variable**	**Model 1**		**Model 2**		**Model 1**		**Model 2**	
	**(1)**	**(2)**	**(3)**	**(4)**	**(5)**	**(6)**	**(7)**	**(8)**
**Likes**								
Style	−0.5564^*^ (0.115)	−0.5029^*^ (0.120)	−0.3936^*^ (0.081)	−0.3575^*^ (0.084)	−3.7726^*^ (0.413)	−3.6292^*^ (0.389)	−0.7395^*^ (0.213)	−0.7181^*^ (0.220)
Special		−0.0555 (0.059)		−0.0923^*^ (0.038)		−0.3796^*^ (0.084)		−0.1383^*^ (0.065)
Style × Special		0.2387^*^ (0.085)		0.2437^*^ (0.065)		0.5940^*^ (0.161)		0.3258^*^ (0.101)
General		0.0201 (0.040)		0.0109 (0.027)		0.1284 (0.067)		0.0392 (0.048)
Style × General		−0.1556^*^ (0.060)		−0.1170^*^ (0.043)		−0.2485^*^ (0.125)		−0.1589^*^ (0.053)
Other variables	Yes	Yes	Yes	Yes	Yes	Yes	Yes	Yes
Cons.	6.6422^*^ (0.741)	6.7913^*^ (0.755)	5.9414^*^ (0.525)	5.9590^*^ (0.523)	16.378^*^ (2.098)	15.646^*^ (2.118)	6.1782^*^ (0.651)	6.2470^*^ (0.631)
Observations	2,410	2,410	2,410	2,410	2,357	2,357	2,412	2,412
(Pseudo) R^2^	0.0958	0.0962	0.8145	0.8162	0.0418	0.0430	0.8119	0.8131
**Comments**								
Style	−0.3119^*^ (0.122)	−0.2958^*^ (0.131)	−0.2999^*^ (0.092)	−0.2697^*^ (0.095)	−2.8782^*^ (0.435)	−2.6945^*^ (0.384)	−0.5837^*^ (0.231)	−0.5761^*^ (0.241)
Special		−0.2786^*^ (0.080)		−0.1615^*^ (0.044)		−0.6146^*^ (0.112)		−0.2182^*^ (0.064)
Style × Special		0.5049^*^ (0.121)		0.3134^*^ (0.074)		0.8090^*^ (0.192)		0.4163^*^ (0.103)
General		0.0106 (0.056)		−0.0366 (0.030)		0.0417 (0.099)		−0.0082 (0.031)
Style × General		−0.1571^*^ (0.078)		−0.1013^*^ (0.049)		−0.1610 (0.154)		−0.1458^*^ (0.042)
Other variables	Yes	Yes	Yes	Yes	Yes	Yes	Yes	Yes
Cons.	4.6891^*^ (1.067)	4.7281^*^ (1.086)	3.5811^*^ (0.601)	3.5543^*^ (0.596)	15.075^*^ (2.849)	12.598^*^ (2.641)	3.7742^*^ (0.548)	3.8053^*^ (0.505)
Observations	2,410	2,410	2,410	2,410	2,357	2,357	2,412	2,412
(Pseudo) R^2^	0.1192	0.1209	0.7396	0.7440	0.0577	0.0619	0.7369	0.7408
**Collections**								
Style	−0.9326^*^ (0.166)	−0.7981^*^ (0.167)	−0.6769^*^ (0.105)	−0.5552^*^ (0.109)	−4.3628^*^ (0.484)	−4.1620^*^ (0.458)	−1.1730^*^ (0.277)	−0.9931^*^ (0.256)
Special		0.1097 (0.075)		0.0764 (0.050)		−0.2590^*^ (0.112)		0.0546 (0.102)
Style × Special		0.2725^*^ (0.113)		0.2268^*^ (0.085)		0.7084^*^ (0.190)		0.2764^*^ (0.131)
General		0.1056^*^ (0.051)		0.0693^*^ (0.035)		0.1843^*^ (0.081)		0.0975^*^ (0.070)
Style × General		−0.2371^*^ (0.079)		−0.1742^*^ (0.056)		−0.3040^*^ (0.151)		−0.2117^*^ (0.080)
Other variables	Yes	Yes	Yes	Yes	Yes	Yes	Yes	Yes
Cons.	6.4927^*^ (1.072)	6.7749^*^ (1.087)	4.7339^*^ (0.684)	4.8347^*^ (0.680)	15.077^*^ (2.262)	15.235^*^ (2.375)	5.0232^*^ (0.840)	5.1414^*^ (0.838)
Observations	2,410	2,410	2,410	2,410	2,357	2,357	2,412	2,412
(Pseudo) R^2^	0.0905	0.0919	0.7460	0.7495	0.0487	0.0500	0.7450	0.7495
**Shares**								
Style	−0.8520^*^ (0.194)	−0.7135^*^ (0.199)	−0.7105^*^ (0.118)	−0.6014^*^ (0.122)	−4.7306^*^ (0.660)	−4.2964^*^ (0.607)	−1.1583^*^ (0.322)	−0.9978^*^ (0.331)
Special		−0.1228 (0.085)		−0.0776 .056)		−0.5502^*^ (0.126)		−0.1243 (0.089)
Style × Special		0.6152^*^ (0.142)		0.3980^*^ (0.095)		1.0137^*^ (0.242)		0.5070^*^ (0.139)
General		−0.0631 (0.063)		−0.0088 (0.039)		0.0224 (0.103)		0.0158 (0.055)
Style × General		−0.0782 (0.106)		−0.1406^*^ (0.063)		−0.2639 (0.200)		−0.1602^*^ (0.068)
Other variables	Yes	Yes	Yes	Yes	Yes	Yes	Yes	Yes
Cons.	6.0857^*^ (1.344)	6.6393^*^ (1.429)	3.7416^*^ (0.767)	3.7594^*^ (0.764)	20.506^*^ (3.105)	19.126^*^ (3.110)	3.9625^*^ (0.956)	4.0080^*^ (0.921)
Observations	2,410	2,410	2,410	2,410	2,357	2,357	2,412	2,412
(Pseudo) R^2^	0.0959	0.0970	0.7345	0.7372	0.0494	0.0520	0.7333	0.7358

^*^p < 0.05; Robust standard errors are in parentheses () for Columns (1) to (4), Robust standard errors estimated via 5,000 bootstrap replicates are in parentheses () for Columns (5) and (6), Robust standard errors clustered by doctor are in parentheses () for Columns (7) and (8); Other variables include control variables and fixed-effect variables [without individual-fixed effects for Columns (5) and (6)].

### 5.3 Endogeneity checks

Although we controlled for some variables to avoid the interference from confounding factors, endogeneity remained a problem that affected our research. In this regard, we adopted a two-stage instrumental variable approach. The instrumental variable (IV) was constructed as the number of short videos in the style of dialogue among the 10 short videos that are closest in terms of release time to a given doctor-generated short video. From the perspective of relevance, due to the nature of their work, doctors typically adopt a shooting approach in batches when producing short videos, resulting in the consistency of teaching style among short videos released around the same time. Additionally, a doctor may be accustomed to a teaching style, ensuring relative stability in the teaching style among her/his short videos. These factors ensure a strong correlation between the IV and the independent variable. From the perspective of exogeneity, viewer engagement (likes, comments, etc.) with a given short video is unlikely to be influenced by other short videos. Besides, viewers' viewings on the platform typically occur independently, and the platform's recommendation algorithm introduces a certain degree of randomness and individual variability in video exposure. Consequently, the IV is irrelevant to our dependent variables, satisfying the exogeneity condition. In the first stage, the regression coefficients on the IV are significant at the 0.1% level, while the second stage reports results that are basically consistent with our main findings, as shown in Columns (5) to (8) of Table 4.

## 6 Discussion and conclusion

Short videos have emerged as a transformative medium for healthcare professionals to disseminate M&H knowledge to the public. On short video platforms, viewers can accomplish dynamic knowledge acquisition and even sharing, with their engagement serving as crucial feedback for this dynamic process (11, 18, 19). Existing scholarly attention has been devoted to investigating the current state of doctor-generated short videos for M&H knowledge dissemination, and some suggestions have been provided to enhance viewer engagement (20–22). Despite these advancements, previous studies have largely neglected to explore teaching styles, such as monologue and dialogue, in these short videos. Within this context, this study aims to explore the effect of monologue vs. dialogue styles on viewer engagement, including likes, comments, collections, and shares. Additionally, given the importance of the effective alignment between teaching styles and teaching content, this study investigates the moderating roles of knowledge specialization and generality in the effect.

Our findings illustrate that the monologue style can elicit greater viewer engagement than the dialogue style in doctor-generated short videos. The reason may be the discrepancy in viewers' perceived value of these videos with different teaching styles. On the one hand, viewers who watch monologue-style short videos feel more involved. Viewers may consider that the effort of doctors in knowledge dissemination aims to educate them. In contrast, doctors who adopt the dialogue style have the main intention of responding to inquiries of their patients, instead of viewers in front of the screen. On the other hand, viewers may receive more useful knowledge from monologue-style short videos. Monologue-style short videos require doctors to prepare elaborate content for knowledge dissemination, whereas dialogue-style short videos focus on the personalized diagnosis and treatment for patients presented in these short videos. Additionally, knowledge specialization and knowledge generality moderate the effect of teaching styles on viewer engagement. When doctors teach professional knowledge, viewers will perceive less value due to a lack of capability to understand the knowledge, which limits the superiority of the monologue style in terms of viewer engagement. Meanwhile, when knowledge has a general scope of application, viewers find these short videos more helpful. Accordingly, an increase in knowledge generality heightens the advantage of the monologue style.

### 6.1 Theoretical implications

Our study makes several theoretical contributions. First, short videos have quickly occupied the forefront of people's daily access to M&H knowledge, which has attracted scholarly attention to the improvement of doctor-generated short videos (1, 19, 22). In this aspect, this research extends the literature by examining the role of teaching styles in viewer engagement. Furthermore, we advance the understanding of the mutual influence between teaching methods and teaching content in knowledge dissemination. While previous research has proposed that the results of communication hinge on communication styles in educational activities, many studies have overlooked the importance of communication content (27–29). In the context of M&H education, we show that the monologue style can be more effective for doctors to impart knowledge to the masses than the dialogue style can, particularly when teaching content relates to low-professional knowledge or common health issues. Third, we enhance insight into viewer engagement in short videos. Previous studies have explored the motivations and influencing factors behind viewer engagement, based on theories including aesthetic theory, flow theory, signal theory, and play theory (11, 18, 70). In this study, we jointly use SET and HSM to explain the intrinsic mechanism and posit that viewer engagement is driven by a sequential process from information content to perceived value and then to behavioral feedback, which is mapped to teaching styles, teaching content, and viewer engagement.

### 6.2 Practical implications

Healthcare professionals, such as doctors, short video platform designers, and healthcare departments, can apply our findings in practice. Although an increasing number of doctors have disseminated M&H knowledge to the public, their achievements vary widely: some short videos receive thousands of likes and frequent comments, while others barely elicit viewer engagement. We find that this phenomenon is associated with doctors' choice of teaching style. Healthcare professionals should adopt the monologue style rather than the dialogue style, as it contributes to the improvement in viewers' perceived value. Given that the monologue style may require more time and effort in the creation process, we suggest that it's best for them to design monologue-style short videos when planning to spread basic professional knowledge or explain common health issues. Moreover, although we encourage doctors to use visual and auditory elements in short videos to attract viewers, we remind them to maintain focus on knowledge content. On the one hand, the use of these peripheral elements does not equate to enhanced M&H education. On the other hand, we discover that only a few elements play a positive and significant role in viewer engagement.

Platform designers should create accurate viewer profiles to identify which medical knowledge and health issues viewers are commonly willing to understand and how receptive they are to such information. By doing so, they can provide viewers with short videos tailored to their needs for M&H knowledge. Given that creating attractive short videos requires significant time and effort, platform designers can supply templates for doctors or develop AI-assisted features on the platforms. Healthcare departments can incentivize healthcare professionals to participate in knowledge dissemination by, for example, linking viewer engagement with short videos to doctors' medical service performance. Notably, it is inappropriate to directly use viewer engagement as an assessment standard. Some M&H knowledge is niche and may not generate high viewer engagement, yet it remains meaningful in knowledge dissemination because it is crucial for those who need it.

### 6.3 Limitations

Our study has a few limitations. First, based on cross-sectional observation, we only collected a limited number of existing short videos for analysis, due to the difficulty of manually coding video features. Future research could benefit from incorporating advanced information technologies to enable large-scale data analysis. In addition, we recommend using experimental or longitudinal approaches to rigorously examine causal relationships. Second, as the information content in M&H short videos is highly professional, we invite two clinicians to evaluate knowledge specialization and generality. However, our clinicians are unable to master all M&H topics across departments. As a result, follow-up research should consider involving doctors from a wider range of departments. Third, although monologue and dialogue are the predominant teaching types in doctor-generated short videos, other types like audience addressing and simulated dialogue could have different impacts, which also warrant scholarly attention. Finally, this study is situated in the Chinese context, focusing on short videos published by certified doctors on Douyin. Grounded in Health Belief Model, our findings are shaped by China's healthcare system and cultural background, which may limit their applicability to other national or cultural contexts. From the perspective of Media Richness Theory, being the most feature-rich and widely used platform, Douyin exhibits distinct content characteristics and communication mechanisms, leading to results that are not suitable for some other platforms. Drawing on Persuasion Theory, the professional certification of doctors ensures source credibility, making it difficult to extend the findings to creators without qualifications. As such, this research topic could be extended to other countries, online platforms, and creators.

## Data Availability

The raw data supporting the conclusions of this article will be made available by the authors, without undue reservation.
